# Digital imaging-assisted quantification of H3K27me3 immunoexpression in luminal A/B-like, HER2-negative, invasive breast cancer predicts patient survival and risk of recurrence

**DOI:** 10.1186/s10020-020-0147-5

**Published:** 2020-02-12

**Authors:** Mário Fontes-Sousa, João Lobo, Silvana Lobo, Sofia Salta, Maria Amorim, Paula Lopes, Luís Antunes, Susana Palma de Sousa, Rui Henrique, Carmen Jerónimo

**Affiliations:** 1 Cancer Biology and Epigenetics Group, IPO Porto Research Center (CI-IPOP), Portuguese Institute of Oncology of Porto (IPO Porto), Research Center-LAB 3, F Bdg., 1st floor, Rua Dr. António Bernardino de Almeida, 4200-072 Porto, Portugal; 2 Department of Medical Oncology, Portuguese Institute of Oncology of Porto, Porto, Portugal; 3 Department of Pathology, Portuguese Institute of Oncology of Porto, Porto, Portugal; 4 Department of Pathology and Molecular Immunology, Institute of Biomedical Sciences Abel Salazar – University of Porto (ICBAS-UP), Porto, Portugal; 5 Department of Epidemiology, Portuguese Institute of Oncology of Porto, Porto, Portugal

**Keywords:** Breast cancer, Epigenetics, Histone mark, H3K27me3, Biomarkers, Prognosis

## Abstract

**Background:**

Breast cancer (BC) is a major health concern and better understanding of its biology might improve treatment decisions and patient outcomes. Histone3 Lysine27 tri-methylation (H3K27me3) is a post-translational histone modification frequently associated with altered gene expression. In BC patients, lower H3K27me3 expression has been associated with worse prognosis. We assessed H3K27me3 immunoexpression with digital imaging software assistance, in a cohort of luminal-like BC patients with long-term follow-up time and evaluated its association with clinically relevant endpoints and its clinical usefulness.

**Methods:**

H3K27me3 immunoexpression was assessed, by means of digital-imaging system, in archival tissue samples of 160 luminal A/B-like HER2-negative invasive BC, stages I-III. Survival analysis was performed using Kaplan-Meier and Cox regression. Cases were categorized as ‘low’ or ‘high’ expression based on cut-off defined by receiver operating characteristic (ROC) curve analysis.

**Results:**

The patient cohort showed a median age of 61-years, with a median follow-up time of 11.7 years. Low H3K27me3 expression (below 85% cut-off) was significantly associated with recurrence, both in univariable (HR = 1.99, 95%CI 1.066–3.724) and multivariable analysis when adjusting for grade and age (HR = 1.89, 95%CI 1.004–3.559). A trend for higher risk of death in low H3K27me3 expression BC was observed (*p* = 0.069), reaching statistical significance in younger patients (*p* = 0.021).

**Conclusions:**

H3K27me3 immunoexpression assessed by digital imaging scoring software is an independent prognosis biomarker in luminal-like BC patients and may assist in more individualized adjuvant treatment decisions, thus potentially reducing recurrences after curative-intent treatment, while sparing unnecessary toxicity.

## Background

Breast cancer (BC) is a major public health concern, with an estimated mortality of 23/100,000 women in European countries (Senkus et al. 2015). The majority (approximately 75%) of invasive BC express hormone receptors, being further sub-classified as luminal A or B-like, in the clinical setting, a surrogate for intrinsic molecular subtypes, which display dissimilar prognosis and entail specific therapeutic strategies (Senkus et al. 2015).

Epigenetics, i.e. gene expression alterations without changes in DNA sequence (Egger et al. 2004), include histone post-translational modifications, which may have distinct roles in cancer biology (Lan et al. 2007; Sauvageau and Sauvageau 2010). Trimethylation of lysine 27 of histone 3 (H3K27me3) is amongst those modifications and is specifically related to Polycomb Repressive Complexes 2 (PRC2). In fact, the Polycomb family of genes are epigenetic transcriptional repressors and key regulators of cell fate, involved in cancer stem cell biology (Lan et al. 2007; Sauvageau and Sauvageau 2010). The core of PRC2 harbors methyltransferases (most notably EZH2) which catalyze the trimethylation of histone H3, and participates in control of gene expression patterns (Sauvageau and Sauvageau 2010; Yoo and Hennighausen 2012). Moreover, activation of PI3K/AKT/mTOR signaling, a pathway involved in endocrine-therapy resistance in BC, was suggested to trigger histone H3 tri-methylation (Zuo et al. 2011). Retrospective analysis previously performed in clinical samples suggested that lower H3K27me3 expression was associated with worse prognosis in those patients, and H3K27me3 was also considered a promising therapeutic target (Ribrag et al. 2015; Takeshima et al. 2015; Ko et al. 2016; Yan et al. 2017; Taube et al. 2017).

In this study we aimed to assess the clinical value of H3K27me3 immunoexpression, assisted by digital imaging software, in a cohort of luminal A/B-like BC patients with a long-term follow-up time, while evaluating associations with clinically relevant endpoints, including cancer recurrence and patient survival.

## Material and methods

### Population of the study

A total of 363 post-surgical female BC tissue samples, corresponding to the same number of patients, were identified at the Department of Pathology of Portuguese Oncology Institute of Porto, corresponding to patients diagnosed and treated at our institution between January 1995 and December 2002. All tissue samples were reviewed by an experienced pathologist (blinded to previous classification). We excluded neoadjuvant treated patients (*n* = 24), ‘triple negative’ (*n* = 56) and HER2-amplified/overexpressing (*n* = 26) cancers, as well as stage IV disease (*n* = 7) and cases with insufficient information/non-assessable material (*n* = 90). Therefore, a total of 160 tissue samples with luminal A/B-like, HER2-negative, stages I-III, invasive BC from female patients were considered eligible for analysis. All collected samples were used after informed consent under protocols approved by institutional ethics committee (Comissão de Ética para a Saúde, IPOPFG_CES-369-2017). Individual clinical files were consulted to retrieve relevant clinical information, complemented by electronic clinical file and cancer registry, whenever possible.

### Definition of clinicopathological variables and endpoints

Data was presented in accordance with recommendations for tumor biomarker prognostic studies (McShane et al. 2006).

Positive hormonal receptor BC was defined as cases expressing Estrogen or Progesterone receptor (PgR) ≥ 1% of neoplastic cells as per international guidelines (Hammond et al. 2010) and HER2 status assessment was carried out according to standard recommendations (Wolff et al. 2013).

Luminal A or B are intrinsic subtypes of BC, that have specific clinicopathological surrogate definitions in the clinical practice (Senkus et al. 2015), namely ‘Luminal A-like’ are ER-positive, HER2-negative, Ki67 low, PgR high and Low-risk molecular signature (when available); on the other hand, ‘Luminal B-like (HER2-negative)’ are ER-positive, HER2-negative, and either Ki67 high or PgR low and High-risk molecular signature (when available).

Results are not presented per stage, since staging criteria have varied along the years and, thus, more objective variables were used preferably. Regarding T stage definition, the cases were classified according to Union for International Cancer Control (UICC) / American Joint Committee on Cancer (AJCC) manuals from the 4th edition through the 6th, in which T stage remained generally consistent (T1 ≤ 2 cm; T2 > 2 cm but ≤5 cm; T3 > 5 cm; T4 any size with extension to chest wall or skin, including inflammatory carcinoma of the breast). These definitions remained almost unchanged even in the most recent 7th or 8th editions (Beahrs et al. 1992; Fleming et al. 1997; Greene et al. 2002; Edge et al. 2010; Amin et al. 2017). N staging has actually varied, so pathological node positive cases (pN+) were further specified if less than or equal to vs. more than 4 metastasized loco-regional nodes.

Recurrence was defined as evidence of loco-regional and/or distant disease over 4 months from diagnosis and after curative-intent surgical treatment. Early recurrence was considered when the event occurred ≤5 years within surgery date and late recurrence if > 5 years from surgery date. Endocrine-treatment resistance was clinically classified according to current international consensus guidelines for advanced breast cancer (ABC 4) (Cardoso et al. 2018).

### H3K27me3 immunoexpression assessment

Expression of H3K27me3 was determined by immunohistochemistry (IHC) in formalin fixed paraffin embedded (FFPE) tissues. A pathologist, blinded to clinicopathological variables, selected a representative block and respective invasive BCC areas (excluding in situ and other non-invasive lesions) in each sample for subsequent H3K27me3 immunostaining analysis. Antigen recovery was performed with pre-heated citrate buffer (pH 6.0) in microwave oven at 700 W (20 min) and endogenous peroxidase activity was blocked by 0.6% hydrogen peroxide. Unspecific reactions were blocked with protein block from Novolink™ Polymer Detection System (Novocastra, Newcastle, UK; 5 min at room temperature). Four-micrometer-thick sections were incubated overnight at 4 °C with the primary antibody tri-methyl-histone H3 (Lys27) rabbit monoclonal (C36B11, dilution 1:1500; Cell Signaling Technology, Danvers, MA, USA). Diaminobenzidine was used as chromogen, followed by hematoxylin counterstaining. Colon adenocarcinoma tissue, which was previously identified as H3K27me3 positive, was used as positive control. Negative control consisted on the omission of the primary antibody.

GenASIs™ software, a digital image IHC scoring system, was used for immunoexpression assessment. Only nuclear staining was considered. A customized profile from positive control was used. Two pre-specified conditions were considered for each assessment: ≥ 5 frames analyzed/case and ≥ 3000 cells analyzed/case, in order to increase the reproducibility of the results and to overcome tumor heterogeneity. Regarding the pre-specified conditions, a median of 6 frames were analyzed/case (range 5–10), with a total of 975 frames throughout the study; and a median 3414 cells were analyzed/case (range 3015–5292), with a total of 546,249 cells analyzed throughout the study.

### Statistical analysis

For statistical analysis SPSS® version 25.0 was used. Associations between categorical variables were assessed using Chi-square test. The optimal H3K27me3 immunoexpression cut-off (to categorize cases as ‘low expression’ or ‘high expression’) was determined using ROC curve analysis. In brief, a graph was constructed considering the highest value of the sum between sensitivity and specificity, and the highest value was selected as cut-off. A time-dependent ROC curve was also built to assess the prognostic value of H3K27me3. Biomarker expression was then dichotomized using as cut-off the value that maximized sensitivity and specificity at 15 years of follow-up. Multivariable analysis was performed using Cox regression model. The log-rank test was used to compare survival between groups in Kaplan–Meier survival curves. All *p*-values were two sided and *p* < 0.05 was considered statistically significant.

## Results

### Clinicopathological characteristics of the cohort

The detailed clinical and pathological characterization of the patients included in this study are depicted in Table 1. Luminal B-like and grade 2 invasive carcinomas predominated. Axillary lymph node metastization was found in over 50% of cases. Most patients received adjuvant endocrine treatment, but also included adjuvant chemotherapy and radiotherapy.

**Table 1:** Clinicopathological features of the general cohort population

Variables	N	%
Luminal subtypes		
A-like	66	41.3
B-like	94	58.8
IDC (versus others)	133	83.1
Grade		
G1	22/158	13.9
G2	81/158	51.3
G3	55/158	34.8
pT Stage		
pT1	55/148	37.2
pT2	87/148	58.8
pT3-p/cT4^a^	6/148	4.0
pN Stage		
pN-	68/153	44.4
pN+	85/153	55.6
pN+≥ 4 nodes	35/85	41.2
Adjuvant Treatment		
Adjuvant CT	63/100	63.0
Adjuvant RT	117/143	81.8
Adjuvant ET	128/132	97.0
Adjuvant TMX only	93/128	72.7
Adjuvant TMX + AI	34/128	26.6
Adjuvant TMX + GOS	1/128	0.8
Recurrence	42	26.3
Early recurrence	22/42	52.4
ET-resistant recurrence	24/42	57.1
Systemic recurrence	33/42	78.6
Death	35	21.9

*n* = 160 unless otherwise specified. ^a^T3 and T4 cases analyzed conjointly due to low N (see text for further details) *Abbreviations*: *AI* Aromatase inhibitor, *CT* Chemotherapy, *ET* Endocrine treatment, *GOS* Goserelin, *IDC* Invasive ductal carcinoma, *RT* Radiotherapy, *TMX* Tamoxifen

### IHC H3K27me3 expression evaluation

**Fig. 1:**
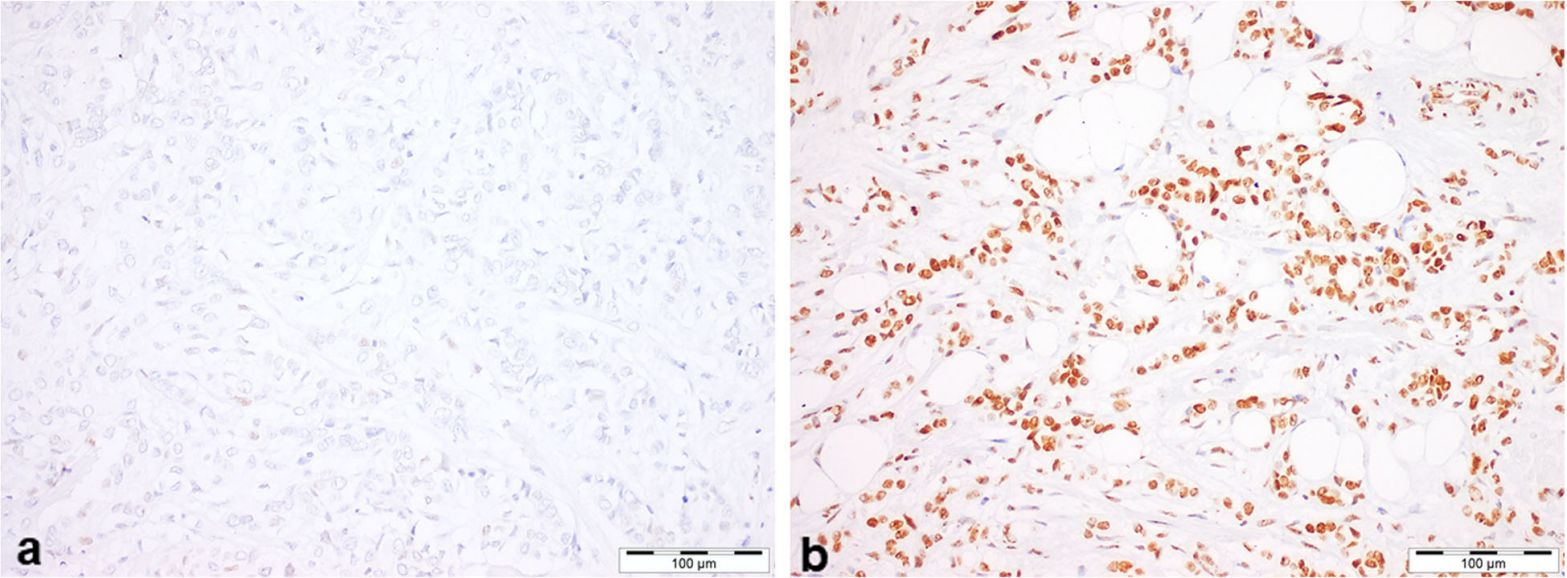
Representative image of H3K27me3 immunoexpression in breast cancer tissue samples. **a** low expression score and **b** high expression

The H3K27me3 nuclear staining varied from 0% (complete lack of H3K27me3 nuclear staining, Fig. 1a) and strong nuclear staining in all analyzed cells (H3K27me3 expression digital software read of 100%, Fig. 1b). The median H3K27me3 expression was 87.2% (range 3.3–99.9%).

The selected cut-off value was 85.3% (Additional file 1: Figure S1), as previously indicated. This value was rounded-up to the nearest integer, i.e., 85% (< 85% ‘low expression’ and ≥ 85% ‘high expression’) for practical purposes. Additionally, time-dependent ROC curve analysis encompassing the 15-year follow-up exhibited a balanced specificity/sensitivity ratio over time (Additional file 1: Figure S2).

### H3K27me3 expression and recurrence and death risks

**Fig. 2:**
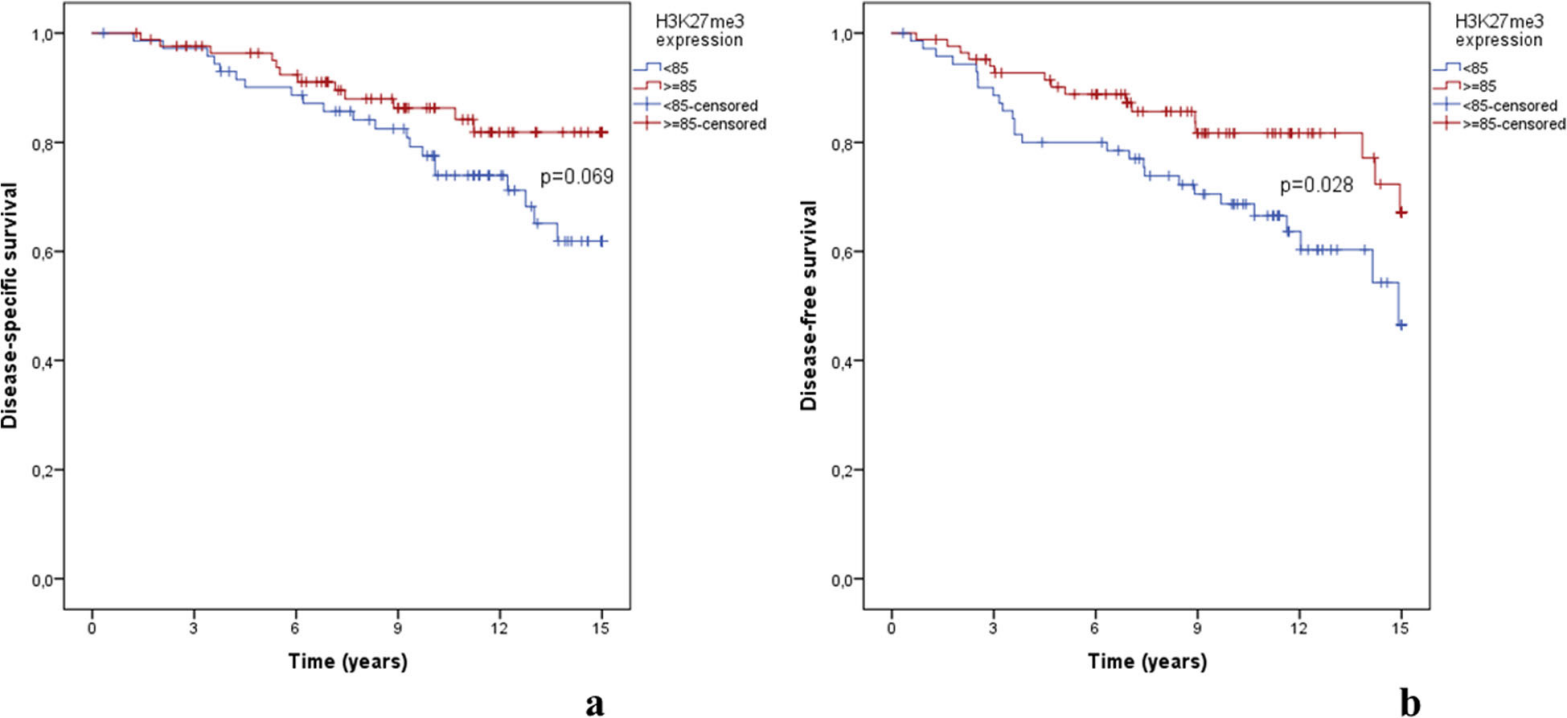
Kaplan–Meier analysis (censored at 15 years of follow-up) for **a** disease-specific survival and **b** disease-free survival, both according to H3K27me3 expression using 85% cut-off

The median follow-up time for the whole cohort was 11.7 years. Disease-specific survival (DSS) was lower for patients with H3K27me3 ‘low expression’, although not statistically significant (*p* = 0.069) (Fig. 2a). DSS at 5-, 10- and 15-years was respectively 90.1, 77.6 and 61.9% for the “low expression group” and 96.3, 86.2 and 81.9% for the “high expression group”. Disease-free survival (DFS) was significantly lower for H3K27me3 “low expression” group (*P* = 0.028) (Fig. 2b). DFS at 5-, 10- and 15-years was 80.0, 68.7 and 46.5%, respectively, for the “low expression” group and 90.1, 81.7 and 67.2%, respectively, for the “high expression” group.

Forty-two patients (26.3%) recurred within 15 years of follow-up. The median H3K27me3 expression in this subgroup was 81.1% (range 8.7–99.7%).

Cases that were scored as “low H3K27me3expression” and presented recurrence were considered as “true positive”, those that depicted “high H3K27me3 expression” and did not recur, were considered “true negative cases”. Accordingly, a sensitivity of 61.9%, a specificity of 59.0%, a positive predictive value of 35.1% and a negative predictive value of 81.2% were obtained considering H3K27me3 expression < 85% as cut-off for predicting recurrence within 15-years (Table 2).

**Table 2:** Specificity and Sensitivity for predicting recurrence and death considering the H3K27me3 85% cut-off

	TP (N)	FP (N)	TN (N)	FN (N)	Sensitivity (%)	Specificity (%)	PPV (%)	NPV (%)
Recurrence	26	48	69	16	60.5	59.0	35.1	80.2
Death	22	52	73	13	63.6	58.3	28.4	86.0

*Abbreviations*: *TP* True Positive, *FP* False Positive, *TN* True Negative, *FN* False Negative, *PPV* Positive Predictive Value, *NPV* Negative Predictive Value

No significant statistical associations were found between H3K27me3 expression and clinicopathological variables (Table 3), thus supporting H3K27me3 expression as a statistically independent prognostic factor.

**Table 3:** Association of H3K27me3 expression (85% cut-off) with clinicopathological features

Variables	H3K27me3 “low” expression (< 85%) N(%)	H3K27me3 “high” expression (≥ 85%) N(%)	*P* value
Number of cases	74 (46.3%)	86 (53.7%)	
Median age			
< 61 years old	37 (50%)	39 (45.3%)	0.557
≥ 61 years old	37 (50%)	47 (54.7%)	
Luminal subtype			
Luminal A-like	31 (41.9%)	35 (40.7%)	0.878
Luminal B-like	43 (58.1%)	51 (59.3%)	
Pathological tumor size			
≤ 2 cm (pT1)	26/71 (36.6%)	29/77 (37.7%)	0.817
> 2 cm (pT2–4)	45/71 (63.4%)	48/77 (62.3%)	
Pathological nodal status			
N0	29/73 (39.7%)	39/80 (48.8%)	0.262
N+ (N1–3)	44/73 (60.3%)	41/80 (51.2%)	
Grade			
1 or 2	43/74 (58.1%)	62/86 (72.1%)	0.069
3	31/74 (41.9%)	24/86 (27.9%)	
Histology			
IDC	62 (83.8%)	71 (82.6%)	0.836
Other subtypes	12 (16.2%)	15 (17.4%)	
Adjuvant chemotherapy			
CT	29/43 (67.4%)	34/57 (59.6%)	0.424
No CT	14/43 (32.6%)	23/57 (40.4%)	
Adjuvant endocrine therapy			
TMX only	45/60 (75%)	48/68 (70.6%)	0.576
TMX + AI	15/60 (25%)	19/68 (27.9%)	

*Abbreviations*: *AI* Aromatase inhibitor, *CT* Chemotherapy, *IDC* Invasive ductal carcinoma, *TMX* Tamoxifen

Cox regression analysis showed that H3K27me3 ‘low expression’ was significantly associated with recurrence, both in univariable (HR = 1.99, 95%CI 1.066–3.724) and multivariable analysis, when adjusting for grade and age (HR = 1.89, 95%CI 1.004–3.559) (Table 4).

**Table 4:** Univariate and multivariate analysis regarding disease recurrence

Variable	Univariable		Multivariable	
	HR	95% CI	HR	95% CI
G3 (vs. G1/2)	2.03	1.102–3.728	1.85	0.995–3.422
Age	0.97	0.942–0.996	0.97	0.941–0.996
H3K27me3<85% (vs. ≥85%)	1.99	1.066–3.724	1.89	1.004–3.559

*Abbreviations*: *G* Grade, *HR* Hazard ratio, *CI* Confidence Interval

**Fig. 3:**
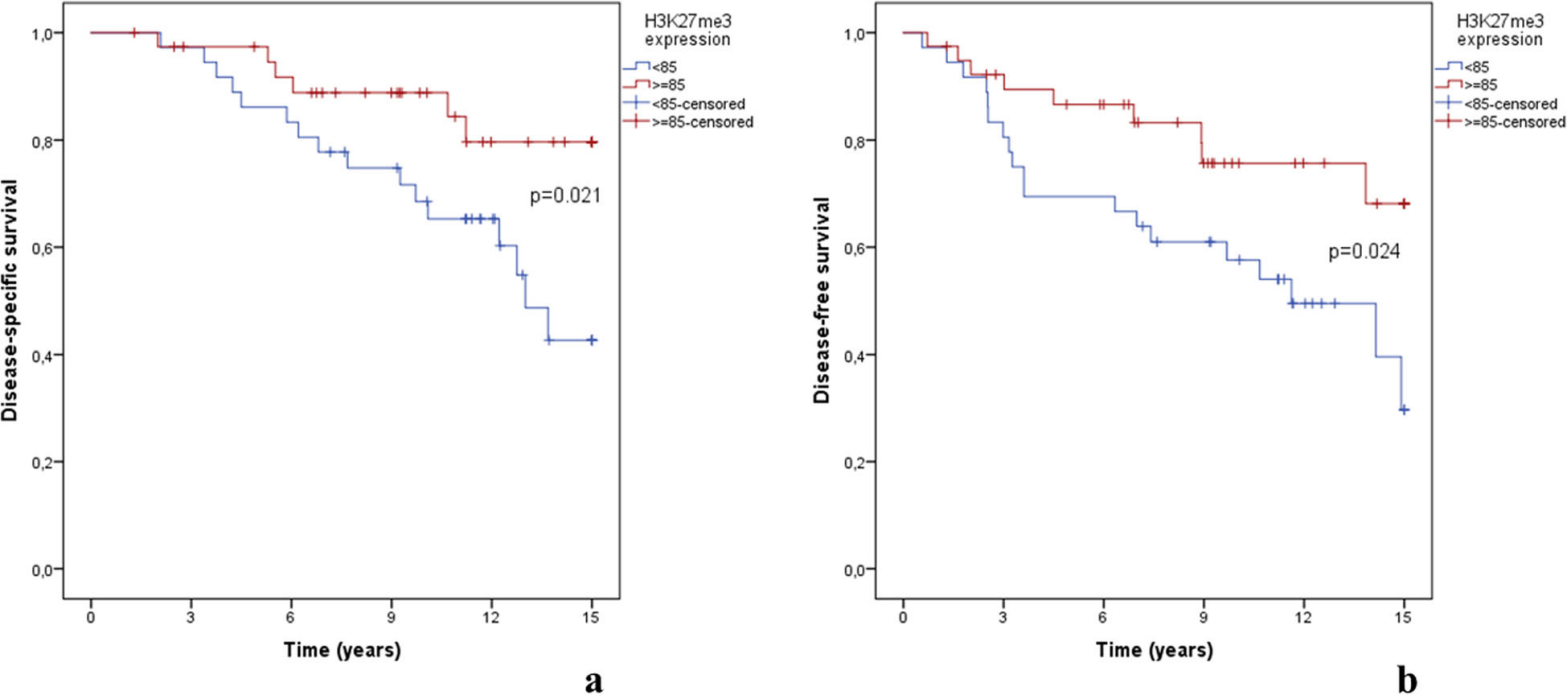
Kaplan–Meier analysis (censored at 15 years of follow-up) in the below the median age population (age < 61 years old; *N* = 76) for **a** disease-specific survival and **b** disease-free survival, both according to H3K27me3 expression using 85% cut-off

During follow-up time, 35/42 (83.3%) of patients in which the disease recurred, eventually died from breast cancer (21.9% of the cohort). In this subgroup, the median H3K27me3 expression was 78.1% (range 8.7–99.7%). In younger patients (below the median of age of 61 years), H3K27me3 ‘low expression” significantly associated with development of disease recurrence and death (*p* = 0.024 and *p* = 0.021, respectively) (Fig. 3).

### H3K27me3 expression and resistance to endocrine-treatment

Overall, no association was found between H3K27me3 “low expression” and early recurrence (*p* = 1.000), systemic recurrence (*p* = 0.199) or endocrine-treatment recurrence (*p* = 0.685). Patients with luminal B-like BC subtype, however, displayed significantly higher risk of endocrine-treatment resistance recurrence (OR 1.905; 95% CI, 1.063–3.425, *p* = 0.009). Conversely, non-G3 tumors depicted significantly lower recurrence risk (OR 0.457; 95% CI, 0.224–0.932, *p* = 0.028).

## Discussion

Herein, we aimed to determine the potential clinical usefulness of H3K27me3 immunoexpression in a defined subset of BC patients, more specifically in stage I-III, luminal A/B-like HER2-negative invasive carcinomas, primarily treated with surgery. Using digital imaging assistance, immunoexpression was quantified in all cases and these were then categorized into “low expression” and “high expression” categories using a statistically defined cut-off. Globally, in this exploratory retrospective study that included 160 invasive BC patients with a median 10-year-plus follow-up, it was found that lower expression of H3K27me3 negatively impacts on prognosis, predicting lower DFS and increased likelihood of recurrence and endocrine resistance, especially in luminal B-like BC.

There is only a limited number of reports on the clinical significance of altered H3K27 methylation in solid tumors. Preliminary findings suggested that lower H3K27me3 expression might predict poor outcome in BC, in a cohort of 142 patients with a median age of 51 years, 43 of which were estrogen receptor (ER) positive (Wei et al. 2008). The median overall survival (OS) was 50 months and H3K27me3 immunoexpression was independently assessed by two pathologists, by eye-ball estimation. The median expression (30%) – much lower than defined in our study - was used as cut-off to determine dichotomic categories of low- and high expression. The reported 5-year survival rate was 46% in cases with low expression of H3K27me3 vs. 72% for high expression (*P* = 0.005). In a study by Holm and colleagues, patients with lower intensity score also revealed shorter DFS (Holm et al. 2012). Moreover, Bae et al. correlated H3K27me3 expression with clinical parameters (Bae et al. 2015), expanding on a previous report (Wei et al. 2008). Of 146 BC patients, 102 were ER positive, with a median age of 46 years (a much younger population when compared to our cohort) and median follow-up time of 6.2 years. Using a score weighting intensity and percentage of positive cells, high H3K27me3 expression associated with longer OS (*p* < 0.001). Healey et al. also showed that H3K27me3 positivity associated with lower grade and luminal A subtype BC, with a follow-up time over 20-years using a dichotomic positivity score with arbitrary 50% expression cut-off (Healey et al. 2014).

Thus, globally, our results are in accordance with previous studies, being consistent in showing that lower H3K27me3 expression is associated with poorer prognosis in ER positive BC. Nonetheless, concerning DSS (a clinical endpoint we favored over OS, since it relates specifically to BC survival), statistical significance was only achieved for younger patients (below the median age). These dissimilarities might be due to distinct populations (e.g., the median age of our cohort was higher) and definitions of hormone-receptor positivity (we used the currently accepted 1% cut-off but other researchers did not). Importantly, the methodology to evaluate H3K27me3 and define a meaningful cut-off also varied considerably: all previously published studies assessed immunoexpression through eye-ball estimation, which is rather subjective and with low reproducibility. Importantly, we evaluated H3K27me3 immunoexpression using a digital-imaging system-assisted software which provides more reliable and reproducible results, providing a more robust basis for translation into the clinical setting. For instance, over 545.000 cells were analyzed by this method in the study. Nonetheless, the global coherence of results across populations (eastern and western) as well as across assessment methods provide strong evidence of the robustness of our findings.

A potential clinical limitation of our study was the exclusion of locally advanced cancers. Actually, most cases in our cohort were pN+ and still no statistically significant difference was detected considering ‘low-’ or ‘high expression” cases (Table 3). Those advanced cases nowadays are most likely treated with neoadjuvant systemic therapy. We excluded such samples since H3K27me3 expression could be affected by drug exposure and become a confusing factor when interpreting the results. Triple negative cancers were excluded, because we focused on luminal BC cases since they represent the majority of cases in the clinical practice and endocrine treatment resistance recurrence was an endpoint of the study. Also, HER-2 positive BC cases were excluded, because HER2 assessment was not routinely performed when the cohort was initially constituted and thus most patients were not treated according to current guidelines, which would make the results not applicable nowadays. Taken altogether, those conditions might explain the globally favorable prognosis found in our cohort, since it was restricted to luminal subtype BC cases.

A potential clinical use of the assessment of H3K27me3 expression, using the 85% cut-off, if prospectively validated, is that it could be used as a tool for clinical trial design, treatment decisions or individualized follow-up protocol. Specifically, considering that low expression associated with increased risk of recurrence, a patient could be candidate for adjuvant chemotherapy followed by endocrine therapy (vs endocrine therapy only), or adjusted follow-up (to detect early recurrences). We would envision this assessment as an additional element among the more routinely established prognostic markers already in clinical use to define adjuvant therapy intensity (such as Ki67, tumor size or nodal status, among others) and even multigene expression panels already in routine practice. Moreover, H3K27me3 immunostaining is performed as easily as Ki67 (antibodies are commercially available) at any facility, and as we showed, with reliable digital imaging software assistance.

A dedicated previous sub-analysis (Fontes-Sousa et al. 2017) focusing on endocrine-treatment resistance did not disclose an associations between H3K27me3 expression and PI3K/AKT/mTOR signaling pathway, at least in this clinical primary BC setting. Interestingly, *PIK3CA* status did not influence the response, when CDK4 and CDK6 inhibitors, such as palbociclib, with fulvestrant, were administered to metastatic BC patients that progressed on previous endocrine therapy [PALOMA-3 trial (Cristofanilli et al. 2016)]. Alternatively, other unknown endocrine resistance’s pathways might also be implicated.

## Conclusions

In conclusion, quantitative assessment of H3K27me3 immunoexpression in luminal A/B-like HER2 negative BC primarily treated with surgery might provide a valuable ancillary tool to assist in prognostication and definition of the best treatment strategy. These results warrant further validation to confirm the usefulness of H3K27me3 assessment as a clinically relevant tool in the era of precision Oncology.

## Supplementary information

**Additional file 1: Figure S1.** ROC curve for assessing the cutoff point with the highest sum of sensitivity and specificity. **Figure S2.** Time-dependent ROC curve for assessing the cut-off value that maximized sensitivity and specificity at 15 years of follow-up.

## Data Availability

All data generated or analyzed during this study are included in this published article and its supplementary information files.

## Abbreviations

ABC: Advanced breast cancer
AI: Aromatase inhibitor
AJCC: American Joint Committee on Cancer
BC: Breast cancer
CI: Confidence Interval
CT: Chemotherapy
DFS: Disease-free survival
DSS: Disease-specific survival
ER: Estrogen receptor
ET: Endocrine treatment
FFPE: Formalin fixed paraffin embedded
FN: False Negative
FP: False Positive
G: Grade
GOS: Goserelin
H3K27me3: Trimethylation of lysine 27 of histone 3
HR: Hazard ratio
IDC: Invasive ductal carcinoma
NPV: Negative Predictive Value
OR: Odds ratio
OS: Overall survival
PgR: Progesterone receptor
pN-: Pathological node negative cases
pN+: Pathological positive negative cases
PPV: Positive Predictive Value
PRC2: Polycomb Repressive Complexes 2
ROC: Receiver operating characteristic
RT: Radiotherapy
TMX: Tamoxifen
TN: True Negative
TP: True Positive
UICC: Union for International Cancer Control
