# Supplementary material for: Digital imaging-assisted quantification of H3K27me3 immunoexpression in luminal A/B-like, HER2-negative, invasive breast cancer predicts patient survival and risk of recurrence
Source: Mol Med. 2020 Feb 12;26:22. doi: 10.1186/s10020-020-0147-5 (PMC7017542; doi:10.1186/s10020-020-0147-5)

ADDITIONAL FILE 1

Figure S1 – ROC curve for assessing the cutoff point with the highest sum of sensitivity and specificity

Figure S2 – Time-dependent ROC curve for assessing the cut-off value that maximized sensitivity and specificity at 15 years of follow-up

Figure S1 ROC curve for assessing the cutoff point with the highest sum of sensitivity and specificity

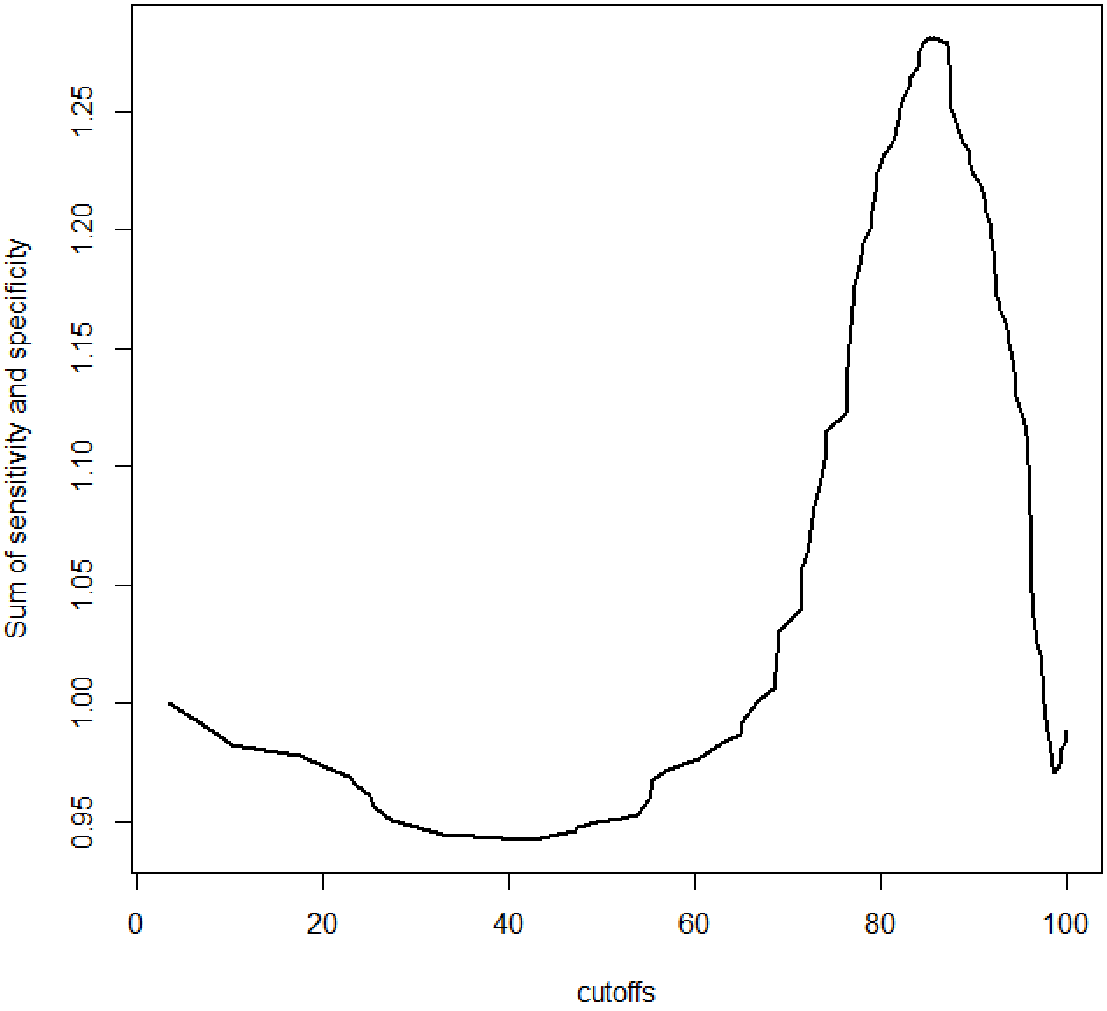

Figure S2 Time-dependent ROC curve for assessing the cut-off value that maximized sensitivity and specificity at 15 years of follow-up

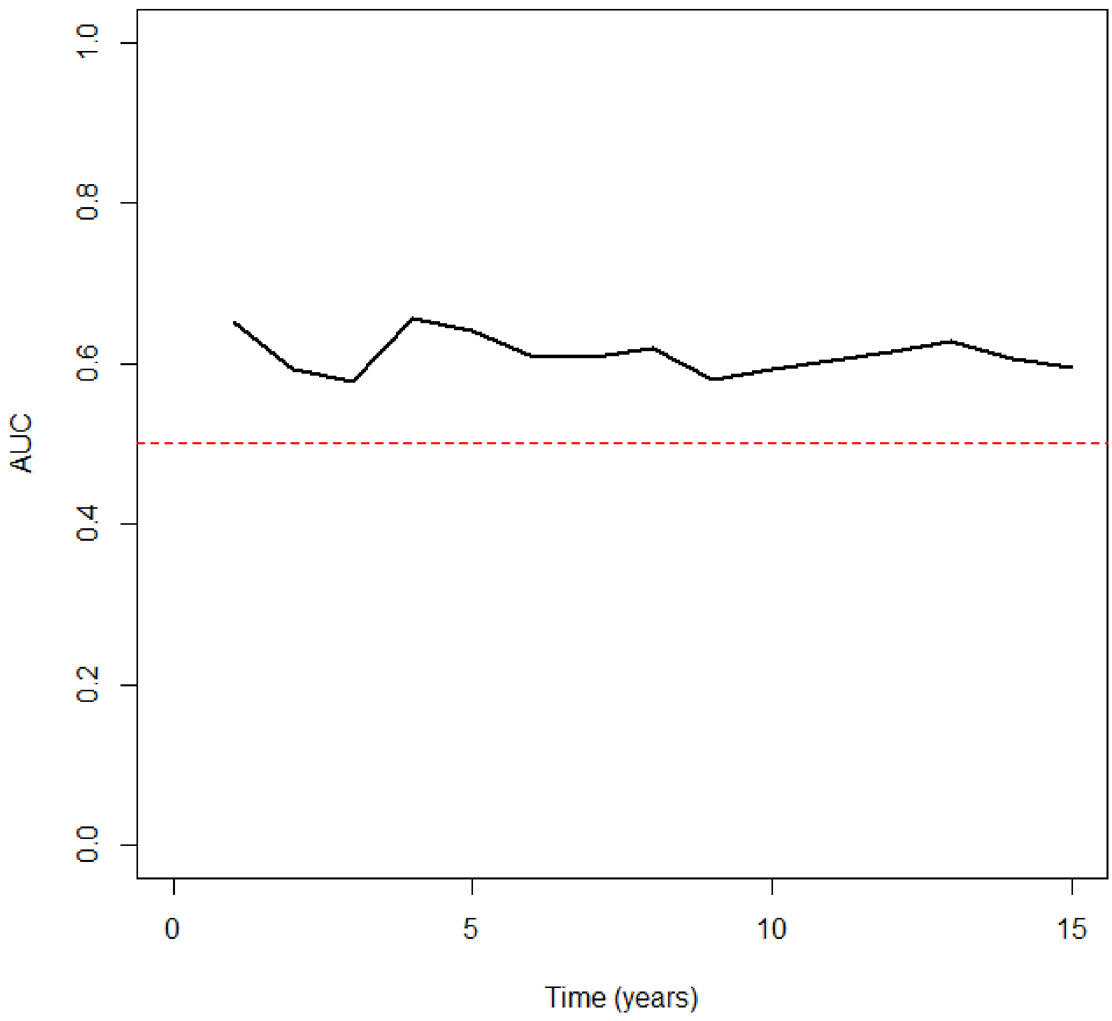

Supplement: Supplementary file 1 — Additional file 1: Figure S1. ROC curve for assessing the cutoff point with the highest sum of sensitivity and specificity. Figure S2. Time-dependent ROC curve for assessing the cut-off value that maximized sensitivity and specificity at 15 years of follow-up. [file 10020_2020_147_MOESM1_ESM.pdf]
